# Supplementary material for: Deletion of the Circadian Clock Gene Per2 in the Whole Body, but Not in Neurons or Astroglia, Affects Sleep in Response to Sleep Deprivation
Source: Clocks Sleep. 2023 Apr 13;5(2):204–25. doi: 10.3390/clockssleep5020017 (PMC10123656; doi:10.3390/clockssleep5020017)
Supplement: Supplementary file 1 [file clockssleep-05-00017-s001.zip › Suppl. Fig/Supplementary Figure Legends.pdf]

Supplementary Figure Legends:

Figure S1. Sleep and activity in baseline during the light and the dark phase. (**A**) Average (± SEM) total time spent asleep over the 12h light and 12h dark period. Values are mean sleep minutes per hour averaged over 7 days of baseline. During the light period, total sleep per hour does not differ between circadian KOs and their respective controls. However, during the dark phase, N*Per2* KO and G*Per2* KO mice slept significantly more. Unpaired, two-tailed t-tests with * p < 0.05, *** p < 0.001, n = 19 *wt* (control $Per2^{Brdm}$), n = 22 $Per2^{Brdm}$, n = 22 *wt* (control T*Per2*), n = 24 T*Per2* KOs, n = 24 *wt* (control N*Per2*), n = 24 N*Per2* KOs, n = 25 *wt* (control G*Per2),* n = 22 G*Per2* KOs. (**B**) Quantification of wheel-running activity. Activity amount of circadian knock-out mice and their controls during the activity and the rest phase did not differ. Unpaired, two-tailed t-tests with * p < 0.05 and n = 5 *wt* (control $Per2^{Brdm}$), n = 6 $Per2^{Brdm}$, n = 6 *wt* (control T*Per2*), n = 5 T*Per2* KOs, n = 4 *wt* (control N*Per2*), n = 6 N*Per2* KOs, n = 18 *wt* (control G*Per2*), n = 15 G*Per2* KOs.

Figure S2. Total time spent asleep in 2h of the light phase before and after SD. Pre-SD the analyzed time window comprised 2 hours in BL from ZT0 to ZT3, the time post-SD encompassed 2 hours starting from sleep onset after sleep deprivation (between ZT6 to ZT7). $Per2^{Brdm}$ mice sleep more than their controls post-SD (**A**). When comparing the pre- and post-SD condition, sleep minutes are increased (or trending) after sleep deprivation in control mice and for $Per2^{Brdm}$ (**A**) and G*Per2* KO mice (**D**) but not for T*Per2* KO (**B**) and N*Per2* KO mice (**C**). These showed already a heightened amount of sleep during pre-SD conditions. 2-way repeated-measures ANOVA with Sidak's *post-hoc* t-test for genotype differences and *post hoc* paired t-tests within genotypes, with * p < 0.05, ** p < 0.001, *** p < 0.001, **** p < 0.0001 and n = 19 *wt*, n = 22 $Per2^{Brdm}$, n = 22 *wt* (control T*Per2*), n = 24 T*Per2* KOs, n = 24 *wt* (control N*Per2*), n = 24 N*Per2* KOs, n = 25 *wt* (control G*Per2*), n = 22 G*Per2* KOs.

Figure S3. Distribution of sleep bout durations comparing pre-SD (2 hours in BL from ZT0 to ZT3, hours 1-2 in figure 1, 2, 3 and 4B) and post SD (the following 2 hours after sleep onset) separately for each genotype and their controls within the light phase. Values are shown as mean (± SEM). Upper panels show the number of bouts per hour of sleep in each sleep bout category (0.5-1min, >1-2min, >2-4min, >4-8min, >8-16min, >16-32min, >32-64min, >64min), lower panels show the percentage of sleep in each of the categories. 2-way repeated-measures ANOVA with Sidak's *post hoc* t-test with * p < 0.05, ** p < 0.001, *** p < 0.001, **** p < 0.0001. (**A**) Controls of $Per2^{Brdm}$ show a reduction of short sleep bouts post-SD, $Per2^{Brdm}$ mice in the short and medium-range length and an increase of time spent asleep in the long sleep bout category, n = 19 *wt*, n = 22 $Per2^{Brdm}$. (B) Like the controls of $Per2^{Brdm}$, controls of T*Per2* KOs have less short sleep bouts after SD, n = 22 *wt,* n = 24 T*Per2* KOs. (C) Controls of N*Per2* KOs have less short to very short sleep bouts and N*Per2* KOs less in the short to medium-short range, n = 24 *wt*, n = 24 N*Per2* KOs. (D) Controls of G*Per2* KOs and G*Per2* KOs display the same pattern: From pre- to post-SD, very short and short sleep bouts are reduced as well as time spent in long sleep bout categories is increased. n = 25 *wt*, n = 22 G*Per2* KOs.

Figure S4. Total time spent asleep in 2h of the dark phase before and after SD. The analyzed time window consisted of 2 hours from ZT13 to ZT15 in either baseline or after sleep deprivation. Only controls reacted to SD by increasing their sleep minutes in this time window (**A-D**). $Per2^{Brdm}$ mice sleep less under both conditions than their controls (pre- and post-SD) (**A**). T*Per2* KO mice sleep less after SD (**B**). For N*Per2* KOs and G*Per2* KOs (**C-D**) no difference to their respective controls are observable in both conditions (baseline and post-SD). 2-way repeated-measures ANOVA with Sidak's *post hoc* t-test for genotype differences and *post hoc* paired t-tests within genotypes, with * p < 0.05, ** p < 0.001, *** p < 0.001, **** p < 0.0001 and n = 19 *wt*, n = 22 $Per2^{Brdm}$, n = 22 *wt* for T*Per2* KOs, n = 24 T*Per2* KOs, n = 24 *wt* for N*Per2* KOs, n = 24 N*Per2* KOs, n = 25 *wt* for G*Per2* KOs, n = 22 G*Per2* KOs.

Figure S5. Distribution of sleep bout durations comparing pre-SD (2 hours in BL from ZT13 to ZT15, hours 13-14 in figure 1, 2, 3 and 4B) and post SD (2 hours during recovery from ZT13 to ZT15, hours 38-39 in figure 1, 2, 3 and 4B) separately for each genotype and their controls within the dark phase. Values are shown as mean (± SEM). Upper panels show the number of bouts per hour of sleep in each sleep bout category (0.5-1min, >1-2min, >2-4min, >4-8min, >8-16min, >16-32min, >32-64min, >64min), lower panels show the percentage of sleep in each of the categories. 2-way repeated-measures ANOVA with Sidak's *post hoc* t-test with * p < 0.05, ** p < 0.001, *** p < 0.001, **** p < 0.0001. (**A**) Within the dark phase, only controls of $Per2^{Brdm}$ display a reaction from pre- to postSD with less short to very short sleep bouts and increased time spent in longer sleep bouts, n = 19 *wt*, n = 22 $Per2^{Brdm}$. (B) Controls of T*Per2* KOs have reduced numbers of short sleep bouts after SD, T*Per2* KOs do not show an altered distribution. Both genotypes show a shift towards longer sleep bout categories in their time spent in each category (individual

comparison significant for T*Per2* KOs in category >4min), n = 22 *wt*, n = 24 T*Per2* KOs. (C) Except for a spike of sleep bout numbers in the short duration category of N*Per2* KOs, distributions are similar for controls and N*Per2* KOs pre- to post-SD, n = 24 *wt*, n = 24 N*Per2* KOs. (D) G*Per2* KOs show a reduction of short sleep bouts post-SD (similar but not as pronounced in controls) and both genotypes have a shift towards longer sleep bout categories of their time spent asleep (significant only for controls, trend for G*Per2* KOs), n = 25 *wt*, n = 22 G*Per2* KOs.

Figure S6. Food intake, water consumption, and ambulatory activity over the course of a day. (**A, B)** Cumulation of food intake in Kcal (1 g food = 3.15 Kcal). $Per2^{Brdm}$ mice starting from ZT8 eat significantly more than controls. (**C, D)** Cumulation of water consumption in ml (1 g = 1 ml). $Per2^{Brdm}$ mice drink significantly more than controls. (**E, F**) Cumulation of ambulatory activity in meters (m). $Per2^{Brdm}$ show less activity in the later dark phase. For all panels: n = 12, repeated measures 2-way ANOVA with Sidak's *post hoc t-test with* * $p < 0.05$, ** $p < 0.01$, *** $p < 0.001$.
