# Supplementary figures and images for: Deletion of the Circadian Clock Gene Per2 in the Whole Body, but Not in Neurons or Astroglia, Affects Sleep in Response to Sleep Deprivation
Source: Clocks Sleep. 2023 Apr 13;5(2):204–25. doi: 10.3390/clockssleep5020017 (PMC10123656; doi:10.3390/clockssleep5020017)

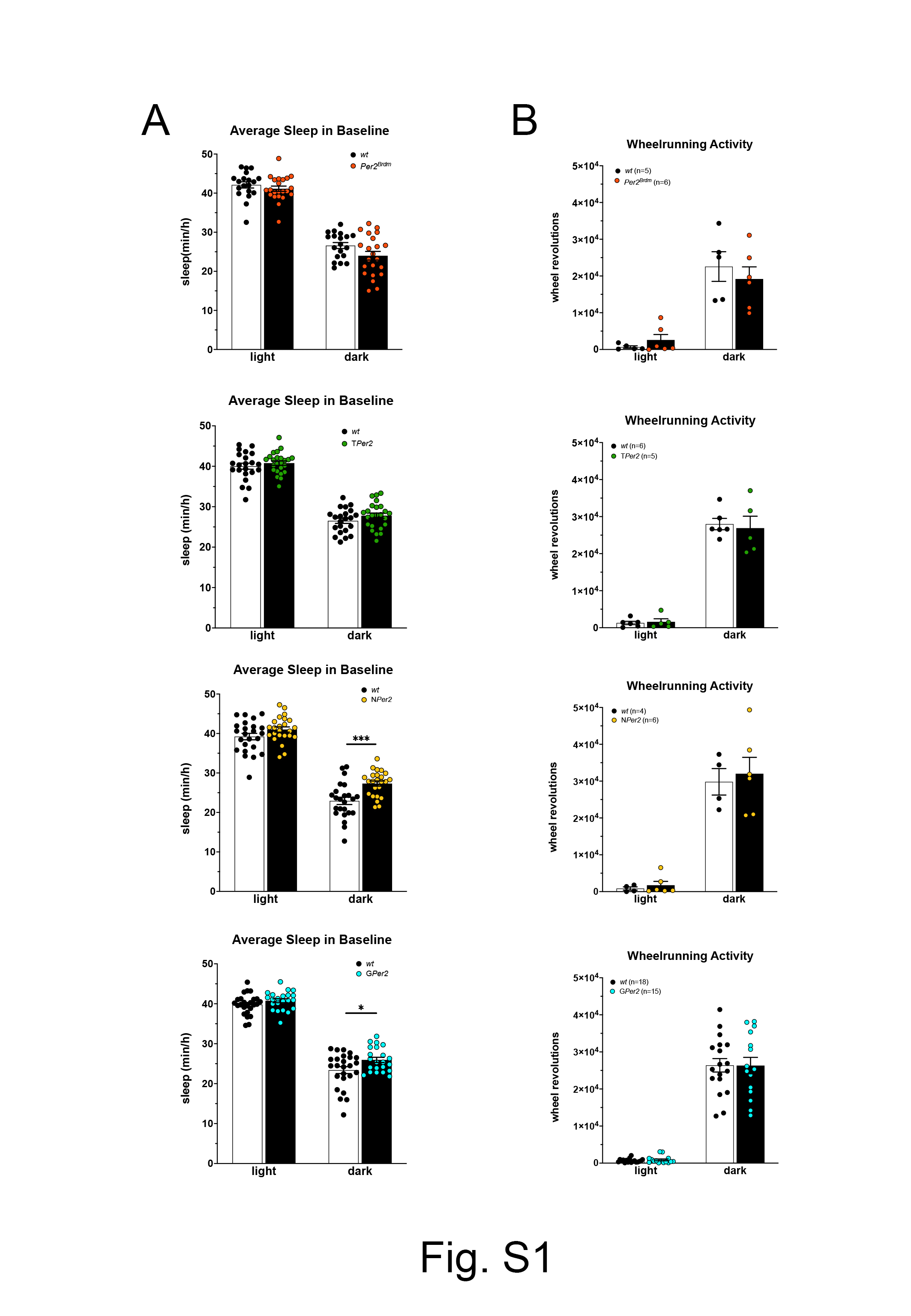

Supplement: Supplementary file 1 [file clockssleep-05-00017-s001.zip › Suppl. Fig/FigS1.png]

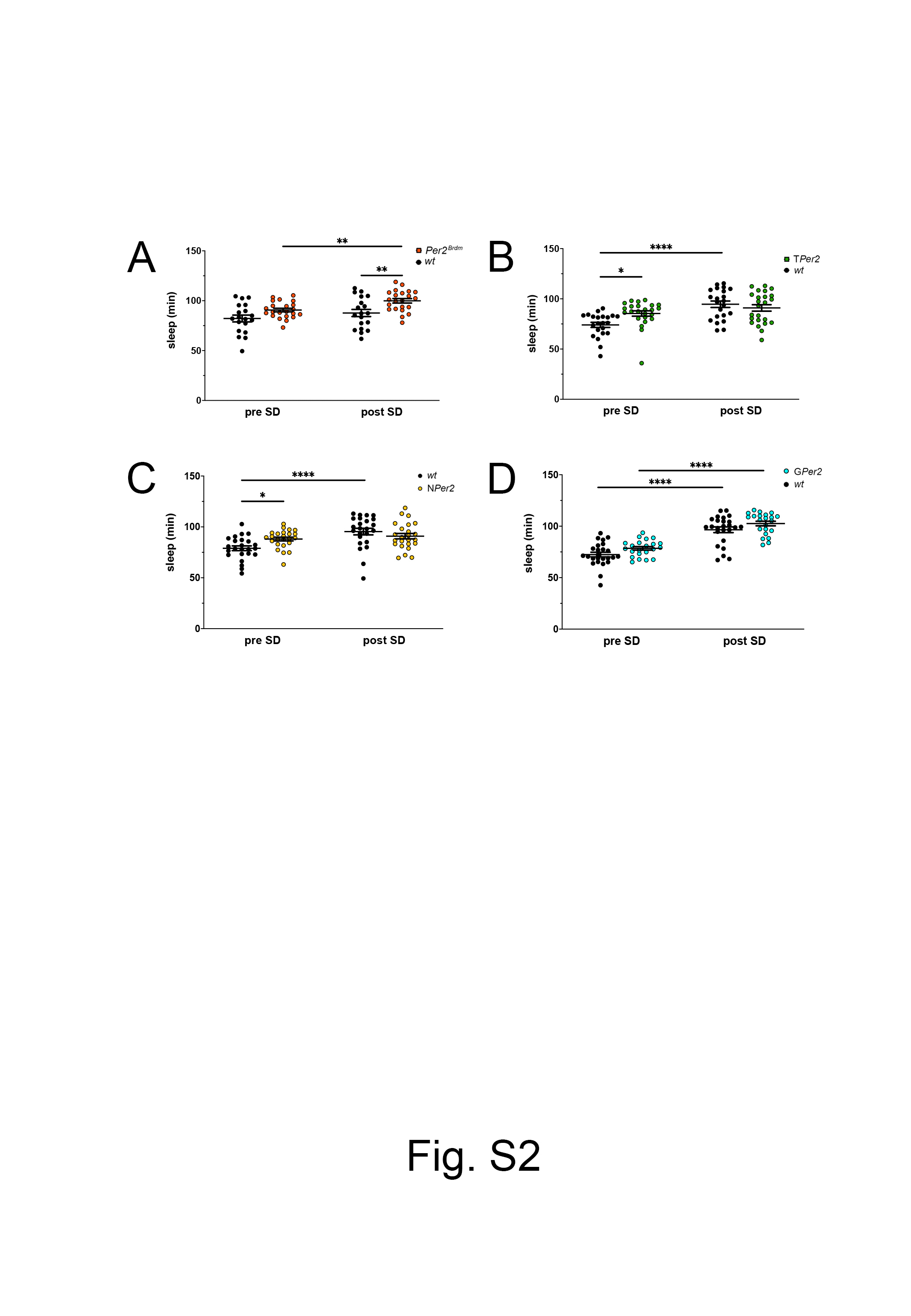

Supplement: Supplementary file 1 [file clockssleep-05-00017-s001.zip › Suppl. Fig/FigS2.png]

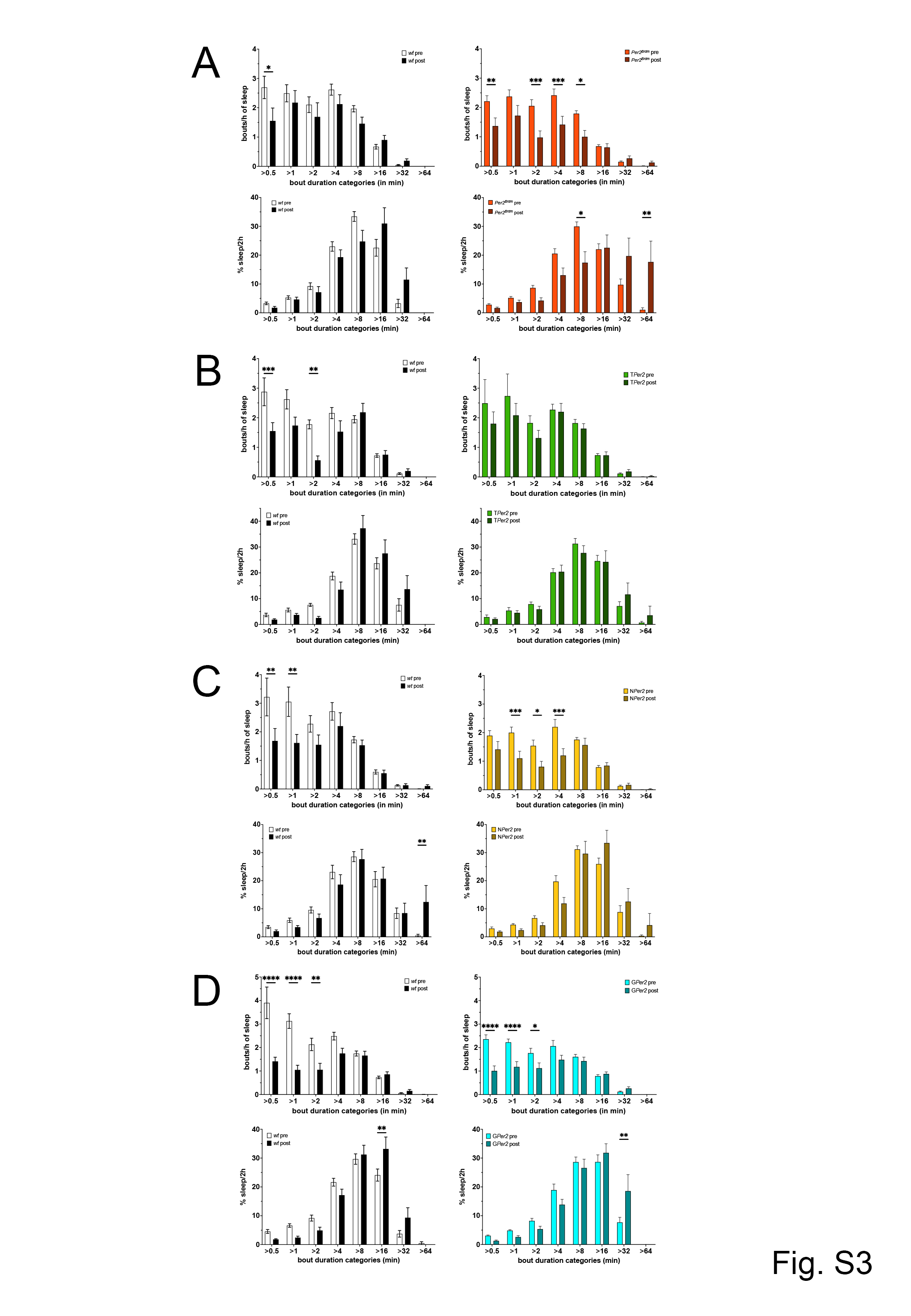

Supplement: Supplementary file 1 [file clockssleep-05-00017-s001.zip › Suppl. Fig/FigS3.png]

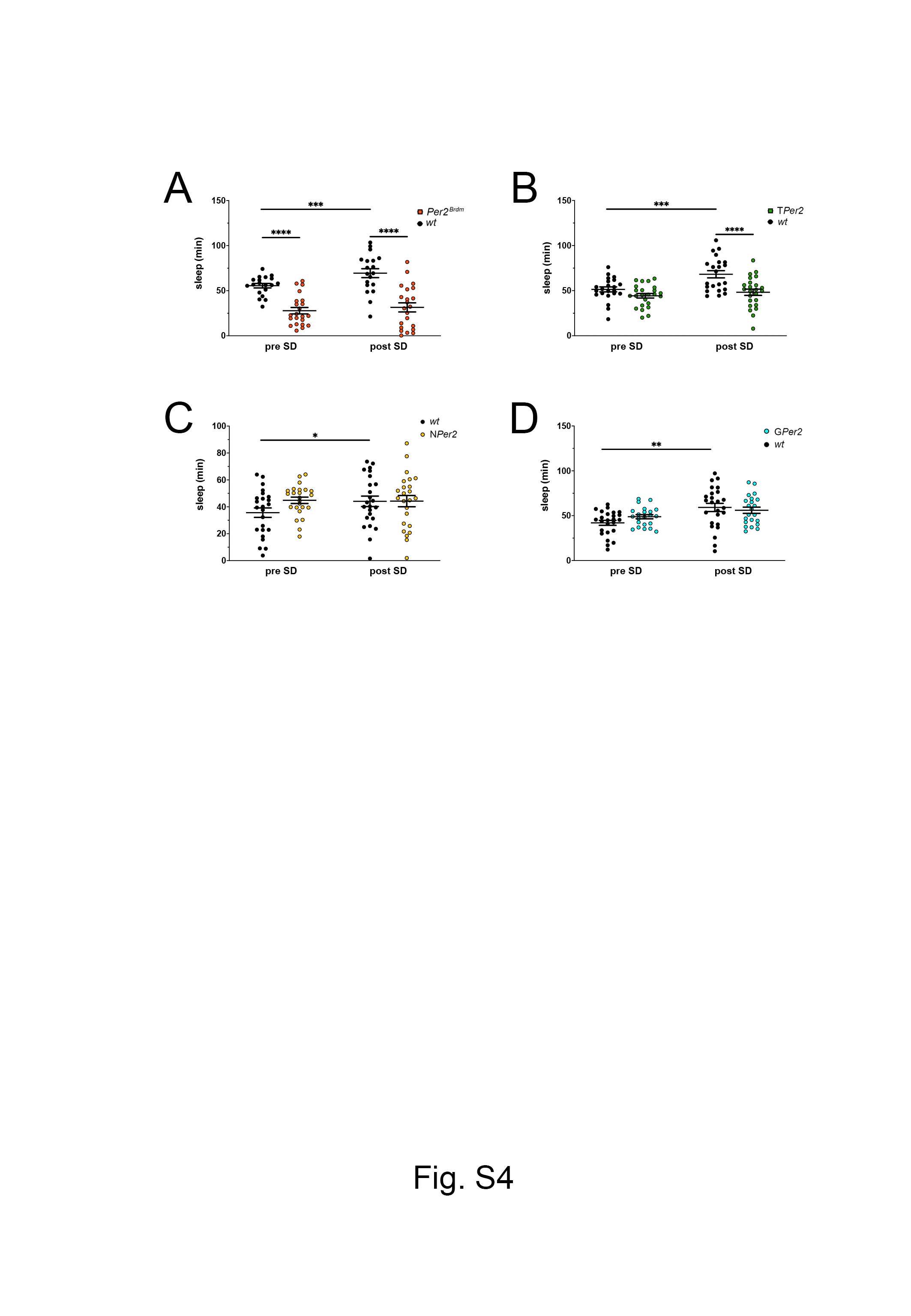

Supplement: Supplementary file 1 [file clockssleep-05-00017-s001.zip › Suppl. Fig/FigS4.png]

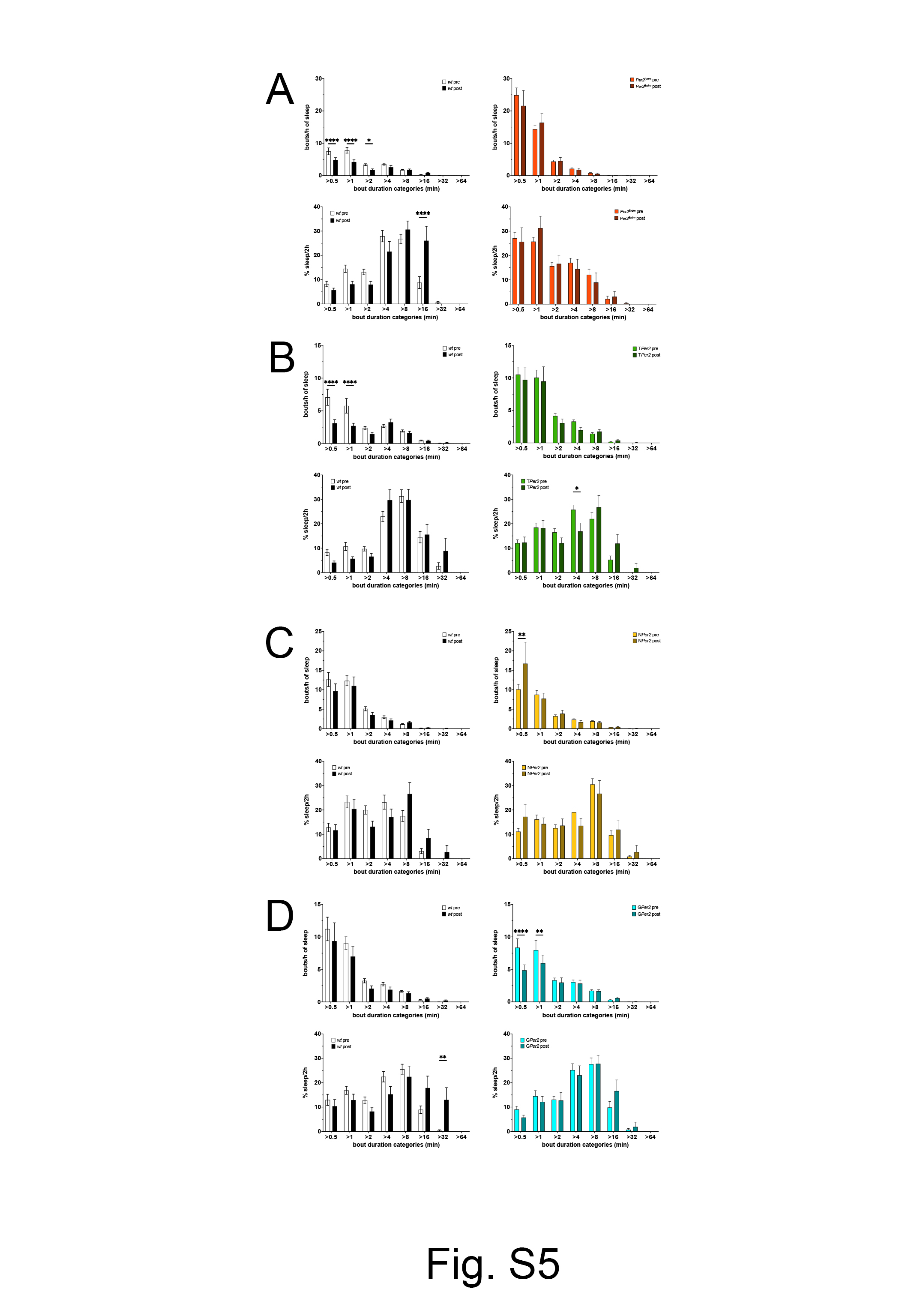

Supplement: Supplementary file 1 [file clockssleep-05-00017-s001.zip › Suppl. Fig/FigS5.png]

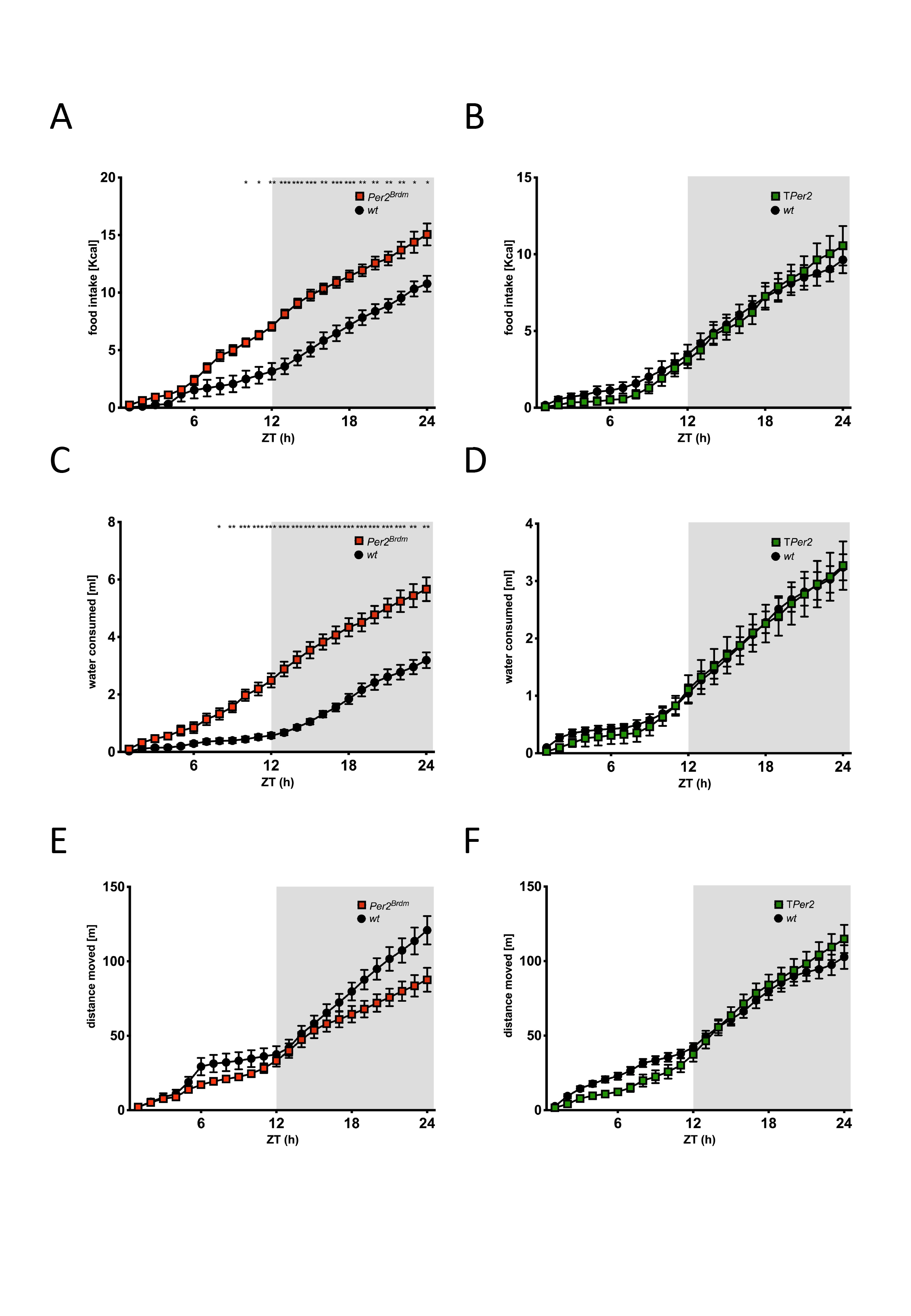

Supplement: Supplementary file 1 [file clockssleep-05-00017-s001.zip › Suppl. Fig/FigS6.png]
